# Extreme image transformations affect humans and machines differently

**DOI:** 10.1007/s00422-023-00968-7

**Published:** 2023-06-13

**Authors:** Girik Malik, Dakarai Crowder, Ennio Mingolla

**Affiliations:** Northeastern University, Boston, MA 02115 USA

**Keywords:** Visual perception, Object recognition, Extreme image transformations, Human-level performance

## Abstract

Some recent artificial neural networks (ANNs) claim to model aspects of primate neural and human performance data. Their success in object recognition is, however, dependent on exploiting low-level features for solving visual tasks in a way that humans do not. As a result, out-of-distribution or adversarial input is often challenging for ANNs. Humans instead learn abstract patterns and are mostly unaffected by many extreme image distortions. We introduce a set of novel image transforms inspired by neurophysiological findings and evaluate humans and ANNs on an object recognition task. We show that machines perform better than humans for certain transforms and struggle to perform at par with humans on others that are easy for humans. We quantify the differences in accuracy for humans and machines and find a ranking of difficulty for our transforms for human data. We also suggest how certain characteristics of human visual processing can be adapted to improve the performance of ANNs for our difficult-for-machines transforms.

**Supplementary Information:**

The online version contains supplementary material available at 10.1007/s00422-023-00968-7.

## Introduction

Driving in heavy snow, rain, a dust-storm or other adversarial conditions impacts the ability of the human visual system to recognize objects. Autonomous systems like self-driving vehicles are even more susceptible to such rarely occurring or out-of-distribution input when interacting with the real world. Object recognition is one of the most fundamental problems solved by primates for their everyday functioning. Humans base their decisions on a wide range of bottom-up and top-down cues, ranging from color to texture to an overall “figure/ground” contour, and on the context that surrounds the object to be recognized (Saisan et al. 2001; Renninger and Malik 2004; Kellokumpu et al. 2011; De Bonet and Viola 1998; Chaaraoui et al. 2013; Al-Ali et al. 2015; Popoola and Wang 2012; Oliva and Torralba 2007; Zhang et al. 2020). Humans combine or seamlessly switch between such cues (Mori et al. 2004; Beleznai and Bischof 2009). These cues help recognize the presence of an “object,” instead of accurately predicting the low-level details about it (e.g., vehicle make, license plate, or text on the rear windshield). The primate visual system is robust to small perturbations in the scene (Zhou and Firestone 2019; Koenderink et al. 2017) and uses sophisticated strategies to recognize objects with high accuracy and confidence.

Artificial neural networks (ANNs) learn to recognize objects with only bottom-up cues like contours, color, texture, etc., allowing them to easily exploit “shortcuts” in the input distribution (Geirhos et al. 2018, 2020) (e.g., a red spherical object is mostly classified as an apple). These shortcuts affect their performance when the objects are distorted by adversarial attacks, limiting their capability to generalize to an out-of-distribution input. ANNs tend to recognize objects both in the presence and in the absence of object structure. This ability also helps them learn from images that appear to be noise to humans (Zhou and Firestone 2019).

Brain regions cannot be directly equated to layers in networks (Yamins and DiCarlo 2016; Dong et al. 2018). While different regions of the brain are primarily responsible for processing different input stimuli (Bear et al. 2020), the visual system processes an object as a “whole” by relying on its contours instead of lower-level features like color. Parts of the objects are assembled, and bounding areas of the key points provide an overall shape for the object (Lee and Mumford 2003; Zhu and Mumford 2007). The reverse hierarchy theory states that humans approach visual classification with a holistic approach, looking at “forest before trees” and then adjust to the lower-level details as needed (Hochstein and Ahissar 2002). Humans calculate the gist of the overall scene before proceeding to do figure-ground segregation and grouping visual objects together (Corbett et al. 2023). Humans also need surprisingly little visual information to classify objects (Ullman et al. 2016). These findings motivate our work — to see how humans and machines perform when the object structure is altered.

To probe the limits of this gap between human and network performance on object classification, we introduce novel image transformation techniques based on what is known in the psychology literature to affect human vision (Edelman et al. 1997; Tarr and Bülthoff 1998; Grill-Spector et al. 2001; Biederman and Cooper 1991; Ferrari et al. 2007), but which go beyond the currently employed techniques of adversarial attacks in machine vision. Our experiments test the limits to which humans and ANNs can withstand these attacks. We further categorize differences between the strategies employed by both to solve tasks with these transforms. We propose a ranking of these attacks based on humans’ ease of solving our recognition task.

***Related work *** Ullman et al. (2016) use “minimal recognizable images” to test the limits of network performance on object recognition and show that they are susceptible to even minute perturbations at that level. Rusak et al. (2020) show that an object recognition model that is adversarially trained against locally correlated noise improves performance. By removing texture information and altering silhouette contours, Baker et al. (2018) show that networks focus on local shape features by shuffling the object silhouettes shown to networks and humans. Baradad Jurjo et al. (2021) try to learn robust visual representations by generating models of noise closer to the distribution of real images. Nguyen et al. (2015) generate images using evolutionary algorithms and attack the networks pretrained on datasets like Imagenet (Deng et al. 2009).

Geirhos et al. (2019) found that Imagenet (Deng et al. 2009) pretrained ResNets (He et al. 2016) recognize the textures in objects with a high accuracy and minimum attention to the segmentations—a dog with the texture of elephant is recognized as an elephant by networks, but is recognized as a dog by humans. Scrambled images do not affect the networks very much (Gatys et al. 2017; Brendel and Bethge 2019), until low-level features are affected (Yu et al. 2017; Ballester and Araujo 2016). Tolstikhin et al. (2021) show the use of patches with multi-layer perceptrons can yield performance rivaling Vision Transformers (ViT) (Dosovitskiy et al. 2021).

Zhou and Firestone (2019) show that while machines can be fooled by adversarial images, humans tend to use their intuition about objects in classifying images that are “totally unrecognizable to human eyes”. They further hint that these intuitions can be used to guide machine classification. Dapello et al. (2021) show that neural networks with adversarial training and general training routines have geometrical differences in their representations in intermediate layers. In Crowder and Malik (2022), authors introduced 5 new transforms with extreme pixel shuffling and found that, barring a few cases, humans perform significantly better than networks on $28 \times 28$ pixel CIFAR100 images. In this work, we show that while the trend holds for some transforms on large $320 \times 320$ pixel Imagenette images, there are significant differences in strategies used by humans and machines to recognize objects.

***Contributions *** Humans and machines use different strategies to recognize objects under extreme image transformations. Humans base their decisions on object boundaries and contours, while networks rely more on low-level features like color and texture.

- We introduce novel image transforms with blocks and image segmentation to simulate extreme adversarial attacks on humans and machines for the task of object recognition.
- We present an extensive study probing the limits of network performance with changes in our transform parameters. We evaluate the performance of ResNet50, ResNet101 and VOneResNet50, as well as 32 human subjects on our transforms.
- We highlight the differences in strategies employed by humans and networks for solving object recognition tasks and present extensive statistical analysis on the performance and confidence of humans and machines on our extreme image transformations.
- We propose a ranking for complexity of transforms (and their parameters) as observed by humans and machines and find that humans recognize objects with contours while machines rely on color/texture, challenging how far network performance is from becoming human-like.

**Fig. 1:**
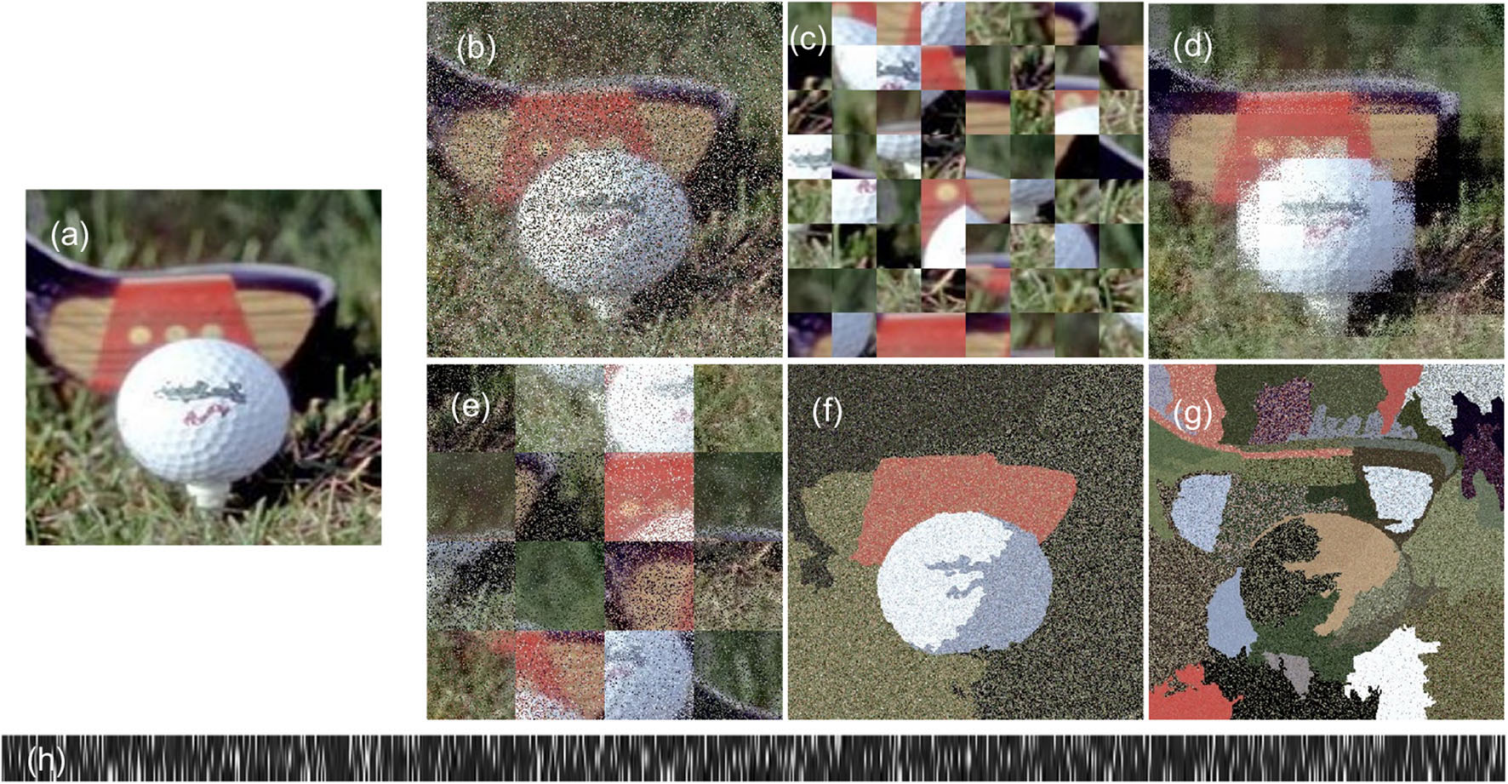
Extreme Image Transformations applied to an Imagenette image of category *Golf Ball*. **a** non-transformed baseline image, **b** Full Random Shuffle with probability 0.5, **c** Grid Shuffle with grid size 40x40, **d** Within Grid Shuffle with block size 40x40 and probability 0.5, **e** Local Structure Shuffle with block size 80x80 and probability 0.5, **f** Segmentation Within Shuffle with 16 segments and probability 1.0, **g** Segmentation Displacement Shuffle with 64 segments, **h** Color Flatten

## Extreme image transformations

A recent trend in deep networks is to meet or exceed the performance of humans on a given task (He et al. 2015; Taigman et al. 2014; Mnih et al. 2015). Papers in the past decades have claimed in silico implementations of primate visual cortex (Douglas and Martin 1991; Wilson and Bower 1991; Bednar 2012). Ullman et al. (2016), however, found that minute changes in the images can significantly impact network performance, while having little or no influence on humans. They showed that human performance remains almost untouched at the scale of minimum recognizable configurations (MIRC). This behavior could be due to networks’ dependence on background and other extra features that they learn to solve tasks. Humans base their decisions on complete and partial presence of features at different scales (Wang and Zhu 2008; Georgeson et al. 2007; Witkin 1987; Lindeberg 2013; Ekstrom and Isham 2017). For example, a silhouette of a zebra can be classified as a horse, but a close look at the ears (not even entire face) might be enough to tell the difference.

Our work asks if humans and machines show a similar response on an object recognition task, without physically breaking down the images into smaller independent images with the atomic representation of the object class (as done in Ullman et al. 2016). We further probe the images at different scales within the blocks and segments by varying block size, the probability of shuffling a pixel, and by interchanging the complete regions with each other.

We introduce seven novel image transformations (Fig. 1), to test the limits of human and machine vision on the object recognition task with distorted image structures. Our transforms can be controlled by three independent variables – (1) **Block size** (or number of segments in case of segmentation shuffles that are described below), (2) **Probability** of individual pixel shuffle and (3) **Moving** blocks or regions to another location or not. Traversing this 3-dimensional space leads to a wide variety of variations in the visual perception of objects for humans and machines (Figure S1). Our transformations can be split into block and segmentation shuffle:

block_shuffle(block_size [#pixels], pix_shuffle_prob [0..1], block_shuffle [0/1]), and

segment_shuffle(segments [#partitions], pix_shuffle_prob [0..1], region_shuffle [0/1])

***Full random shuffle*** moves pixels within the image based on a specified probability (range: [0.0–1.0]), disregarding any underlying structural properties of the image. For a shuffle probability of 0.5, each pixel’s location has a 50% chance of being shuffled, while a shuffle probability of 1.0 moves every pixel around, with the image looking like random noise. Lower probability alters local structure while higher probability alters the global structure of the image. Figure 1b.

***Grid shuffle*** divides the image into blocks of equally sized squares. The divided units are shuffled and rearranged to create an image of the same size as input. Block length is chosen out of [20, 40, 80, 160] pixels. Grid Shuffle alters only the global structure of the image. Figure 1c.

***Within grid shuffle*** divides the image into blocks (similar to Grid Shuffle), but does not shuffle the blocks. Instead, it shuffles the pixels within the blocks with a specified probability. Pixel shuffling within the block is similar to Full Random Shuffle, considering each unit in the block to be an individual image. Grid size and probability of shuffle is in [20, 40, 80, 160] pixels and [0.0–1.0], respectively. Alters only the local structure of the image. Figure 1d.

***Local structure shuffle*** is a combination of Within Shuffle and Grid Shuffle. It divides the image into blocks (like Grid Shuffle), shuffles the pixels within the blocks (like Full Random Shuffle), and further shuffles the positions of the blocks. Alters both global and local structure of the image. Figure 1e.

***Color flatten*** separates the three RGB channels of the image and flattens the image pixels from 2-dimensional $N \times N$ to three channel separated 1-dimensional vectors of length $N*N$ in row-major order. Alters both global and local structure of the image. Figure 1h.

***Segmentation within shuffle*** builds on the grid shuffle paradigm by segmenting the image into regions based on superpixels (Achanta et al. 2010, 2012). The pixels within the region are shuffled with a specified probability in the range [0.0–1.0]. The number of segments is picked from [8, 16, 64]. Figure 1f.

***Segmentation displacement shuffle*** segments the image into regions (8, 16 or 64) based on superpixels (Achanta et al. 2010, 2012). The pixels within each region are shuffled and placed into other regions. The number of pixels in every region can differ significantly prohibiting a smooth displacement when moving pixels from smaller region to a larger region. We solve this problem by re-sampling a number of pixels equal to the difference in number of pixels between larger and smaller region again from the smaller region. We also shuffle them with all the pixels from the smaller region and arrange them in the larger region. Moving from larger to smaller region, we drop the extra pixels. Figure 1g.

***Local vs global manipulations*** It is difficult to precisely categorize transforms into local or global manipulators. Our approach holds that local transforms manipulate the low-level features of the image (not necessarily only texture, but also some borders of the object), while global manipulations alter the overall shape of the object. For example, a Full Random Shuffle with a low probability of say 0.3 can be broadly categorized as a local manipulator, but with a probability of 1.0, the same transformation changes the global structure. Humans are known to easily switch between local and global structures when performing object recognition, while networks generally do not have a way of doing that. To this end, we also rank our transforms based on human accuracy, which favors preservation of global structure, whereas the networks’ rankings tend to rely more on local structure.

## Model selection

We tested ResNet50, ResNet101 (He et al. 2016), and VOneResNet50 (Dapello et al. 2020) for our experiments with baseline (no shuffle) and transformed images. VOneResNet50 was selected for its claims of increasing robustness of Convolutional Neural Network (CNN) backbones to adversarial attacks by preprocessing the inputs with a VOneBlock—mathematically parameterized Gabor filter bank—inspired by the Linear-Nonlinear-Poisson model of V1. It also had a better V1 explained variance on the Brain-Score (Schrimpf et al. 2020) benchmark at the time of our experiments. We chose ResNet50 since it formed the CNN backbone of VOneResNet50 and we wanted to test the contribution of non-V1-optimized part of VOneResNet50. Subsequently, we chose ResNet101 for its high average score on brain-score in terms of popular off-the-shelf models that are widely in use, and for its larger capacity compared to ResNet50.

## Experiments

***Setup: *** We evaluated ResNet50, ResNet101, and VOneResNet50 on the Imagenette dataset [59] against baseline images (without transforms), six shuffle transforms, and Color Flatten transform described in Sect. 2. Imagenette is a subset of 10 unrelated Imagenet (Deng et al. 2009) classes. We used the default train-test split of 9469 training images and 3925 test images from the dataset, distributed over 10 classes. Each image has 3 channels and $320 \times 320$ pixels. We did *not* separate a validation set for network fine-tuning to mimic how humans only see a small subset of objects and then recognize them in the wild, without fine-tuning their internal representations. To further this claim, we also performed 0-shot experiments with Imagenet pretrained networks on images processed with our transforms (please see § S3). We used Imagenette for training and testing, given the models we selected are all trained on the larger Imagenet dataset.

***Training: *** We trained each model on the baseline and transformed images using the default hyperparameters listed in the PyTorch repositories of the respective models. All models were trained for 70 epochs, with a learning rate of 0.1, momentum set to 0.9 and weight decay of $10^{-4}$. For the Grid Shuffle transformation, we used four block sizes – $20 \times 20$, $40 \times 40$, $80 \times 80$ and $160 \times 160$ (dividing image into 4 blocks). For Within Grid Shuffle and Local Structure Shuffle, we used a combination of four block sizes ($20 \times 20$, $40 \times 40$, $80 \times 80$ and $160 \times 160$) with a shuffle probability of 0.5 and 1.0 for each block. For Full Random Shuffle, we used shuffle probabilities of 0.5, 0.8 and 1.0. We used 23 different block transformations.

In the Color Flatten transform, we separated the image channels and flattened the 2D array to 1D in row-major order. We added an additional Conv1D input layer to the networks to process the 1D data.

Humans can base object recognition decisions on the boundaries of objects (Edelman et al. 1997; Tarr and Bülthoff 1998; Biederman and Cooper 1991; Ferrari et al. 2007; Hubel and Wiesel 1962, 1963; Wiesel and Hubel 1963; Hubel and Wiesel 1963; Wiesel and Hubel 1963; Tanaka 1997; Grill-Spector et al. 2001). We wanted to test networks in the same settings by using superpixels to segment objects into varying number of regions and shuffling pixels within/across regions. We trained and tested each of the three models on the segmentation shuffles described in Sect. 2. For Segmentation Displacement Shuffle, we segmented the images into 8, 16 and 64 regions. For Segmentation Within Shuffle, we used a combination of 8, 16 and 64 regions, with a pixel shuffle probability of 0.5 and 1.0. We trained and tested on 9 unique segmentation transformations. All networks were trained end-to-end using only the respective transform (and its hyperparameters), without sharing any hyperparameters across same or different types of transforms.

## Human study

To investigate mechanisms used by humans for solving object recognition task under adversarial attacks and compare it to networks, we ran a psychophysics study with 32 participants on a Cloud Research’s Connect platform. We randomly sampled 3 images from each of the transform-parameter pair to test the subjects, after training them on 11 sample images from Imagenette dataset. We used the same $320 \times 320$ pixel resolution images for both humans and networks and presented all subjects with the same 10 classes to choose from. We also asked the subjects to indicate their confidence about their response on a scale of 1–5. The classes were randomly shuffled on every trial. We gave feedback to subjects after every trial during training phase, but not during the testing phase. We timed their responses on test trials, but they were asked to complete the trials at their own pace. We turned off the timer after every 10 test trials to allow for breaks if needed. We used the same set of three unique images to use with a particular transformation-parameter pair to show to all participants. This means the participants saw the same set of 102 unique images during the entire study. None of the images were repeated during the training or testing phase to avoid learning any kind of biases in object structures for that exact image. For more details about experiment setup, participant filtering and statistical tests, see §S1 and §S2.

## Results

**Table 1:** Test accuracy for models trained on Imagenette dataset with Block transforms

Transform	*P*	Grid size	Accuracy (in %)		
			ResNet50	ResNet101	VOne
Baseline			86.24	85.61	72.2
Full random shuffle	0.5		83.9	83.44	72.22
	0.8		60.66	62.54	49.78
	1.0		47.46	49.3	31.62
Grid shuffle		20$\times$20	84.99	84.46	49.47
		40$\times$40	86.78	86.29	52.28
		80$\times$80	86.29	86.37	70.19
		160$\times$160	85.61	85.2	75.06
Within grid shuffle	0.5	20$\times$20	83.67	83.69	70.93
		40$\times$40	83.11	82.9	72.25
		80$\times$80	83.11	82.9	74.78
		160$\times$160	83.44	83.16	70.78
	1.0	20$\times$20	79.11	78.57	69.45
		40$\times$40	72.51	71.92	67.64
		80$\times$80	65.58	64.2	56.31
		160$\times$160	53.89	54.08	40.61
Local structure shuffle	0.5	20$\times$20	76.89	75.95	58.44
		40$\times$40	81.78	80.74	57.99
		80$\times$80	82.55	82.11	65.55
		160$\times$160	80.66	81.15	60.46
	1.0	20$\times$20	68.18	68.19	49.4
		40$\times$40	66.24	65.22	45.27
		80$\times$80	58.11	57.5	44.43
		160$\times$160	51.36	49.53	36.0
Color flatten			75.67	73.83	59.03

**Table 2:** Test accuracy for models trained on Imagenette dataset with Segmentation transforms

Transform	*P*	Segments	Accuracy (in %)		
			ResNet50	ResNet101	VOne
Segmentation displacement shuffle		8	42.98	38.39	4.54
		16	45.53	45.65	4.01
		64	53.68	53.89	19.97
Segmentation within shuffle	0.5	8	71.17	72.79	4.63
		16	72.95	72.95	4.73
		64	75.51	76.07	4.58
	1.0	8	51.93	48.41	4.91
		16	57.99	48.41	4.91
		64	67.94	67.86	4.75

**Fig. 2:**
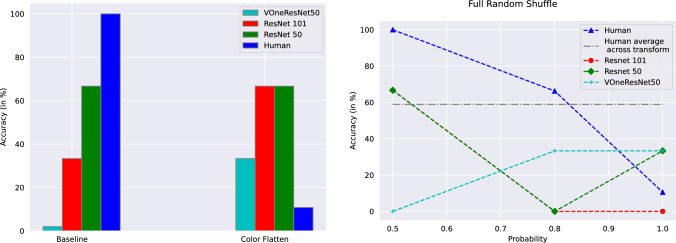
Performance of humans and networks on same images for Baseline and Color Flatten transforms (left) and Full Random Shuffle transform as a function of probability (right). See Table S2 and text for details

ResNet50 performs the best on baseline (without transforms) Imagenette test images, with 86.2% accuracy, followed by ResNet101 and VOneResNet50 (Table 1). The trend is constant across all transforms for VOneResNet50, wherein ResNets on an average perform about 25% better than VOneResNet50. Transformations start reducing the performance of networks. For Full Random Shuffle, the performance decreases by only about 2% with a 0.5 shuffle probability (equal chance of every pixel either moving or staying in the same location), implying that the signal to noise ratio might still be the same as the original image. Increasing the shuffle probability to 0.8–1.0 affects the performance most, reducing to almost half of the original performance. ResNets perform significantly better VOneResNet50. For Grid Shuffle, ResNets stay constant and at par with their baseline performance across all block sizes, while VOneResNet50 suffers from a decrease in block sizes. In case of Color Flatten, our most extreme structure destroying transformation, the performance drops by about 11% for each network compared to their baselines. The networks still perform above chance, implying that recognition is handled independently of the object’s structure.

Changing probability and block sizes together, we find that Local Structure Shuffle is affected more than the Within Grid Shuffle. In case of Within Grid Shuffle, only local structure is altered inside the blocks. The performance trend is reversed compared to the Grid Shuffle, such that an increase in block size reduces performance for shuffle probability of 1.0, but stays constant for a shuffle probability of 0.5. The Local Structure Shuffle alters both the local and global structure of the object. For a shuffle probability of 0.5, the performance seems to be increasing with an increase in block size, given larger block sizes help keep more pixels together during convolutional operations, while a probability of 1.0 reverses that trend, reducing the accuracy with an increase in block size.

Following our experiments about fixed block sizes, we wanted to probe the networks with representations that primates are more comfortable with during object recognition (Edelman et al. 1997; Tarr and Bülthoff 1998; Grill-Spector et al. 2001; Biederman and Cooper 1991; Ferrari et al. 2007; Hubel and Wiesel 1962, 1963; Wiesel and Hubel 1963; Hubel and Wiesel 1963; Wiesel and Hubel 1963). We repeated similar experiments with our segmentation transforms (Table 2). Interestingly, VOneResNet50 suffers the most by this change, dropping the accuracy by over 60% to single digits. For our Segmentation Displacement Shuffle, we found the networks showed an improved performance with a decrease in the size of segments, again implying better performance despite higher structure alterations locally. We observe a similar trend in case of Segmentation Within Shuffle. ResNets show a greater accuracy in this case compared to Segmentation Displacement Shuffle, but with a similar decrease in performance with an increase in shuffle probability. (Please see §S3 for saliency maps of Imagenet pretrained networks on our transforms.)

***Comparison with human responses *** Human subjects show no correlation with the networks’ performance (Tables S2, S3 and S4). The trends in performance are asymmetrical between the two (Fig. 7). Humans perform with a perfect score on baselines and Full Random Shuffle with 0.5 shuffle probability. Human accuracy declines, but is better than networks on a 0.8 shuffle probability case, while it is random at best with a 1.0 shuffle probability (Fig. 2).

On Grid Shuffle, humans show an increase in performance with an increase in block sizes, reaching a perfect accuracy at block sizes 80 and above, a trend similar to networks but at differing accuracies. On Within Grid shuffle with 0.5 shuffle probability, the accuracy only dips for a block size of 80, but remains better than networks otherwise (the networks have a constant performance). With a shuffle probability of 1.0, the performance is much lower than the networks, with a non-monotonic trend (Fig. 4).

**Fig. 3:**
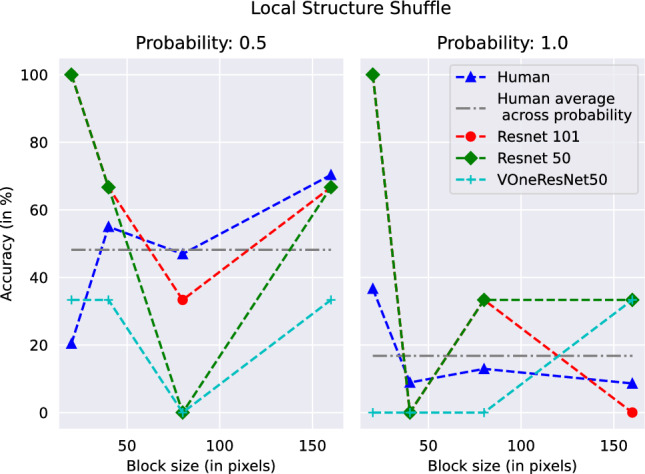
Performance of humans and networks on same images for Local Structure Shuffle with probability 0.5 (left) and probability 1.0 (right) as a function of block size. See Table S2 and text for details

For Local Structure Shuffle with 0.5 shuffle probability, we see a non-monotonic trend with a much lower performance compared to the networks (Fig. 3). The trend remains similar for the 1.0 shuffle probability case, with numbers comparable to Full Random Shuffle 1.0 probability. Color Flatten also affects the human perception to the level of random decision (Fig. 2).

For our segmentation displacement cases, we see that humans consistently perform better than networks, indicative of the human visual system’s reliance on contours for object recognition. When displacing the shuffled pixels across regions, we see human accuracy plummeting to lower than ResNets. When only shuffling within the regions, human performance is very close to the perfect score in the 0.5 shuffle probability case, but takes a hit with the 1.0 shuffle probability case (Fig. 5). The performance in all cases is much higher than that of VOneResNet50 – the network claiming to explain V1 variance.

**Fig. 4:**
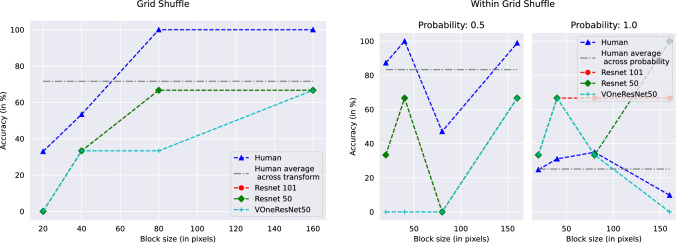
Performance of humans and networks on same images on Grid Shuffle as a function of block size (left); and Within Grid Shuffle (right). The plot for Within Grid Shuffle shows performance on both probabilities as a function of block size. See Table S3 and text for details

**Fig. 5:**
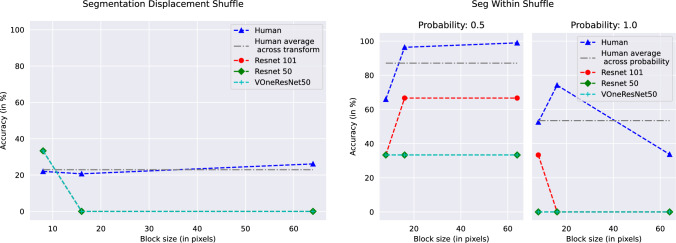
Performance of humans and networks on same images with Segmentation transforms. Segmentation Displacement Shuffle is presented as a function of block size (left); and Segmentation Within Shuffle (right). The plot for Segmentation Within Shuffle shows performance on both probabilities as a function of block size. See Table S2 and text for details

***How different are strategies used for object recognition by humans and machines?*** Humans show a higher performance on certain images compared to machines, while machines show near baseline performance on images that can be classified as noise at best by humans. To answer the question about the strategies employed by humans and machines to solve object recognition task, we evaluated both humans and machines on the same set of images. We additionally asked how confident they were with their decision of selecting the object class present in the image. As expected, the human confidence scores plummeted with an increase in complexity of the transform.

**Fig. 6:**
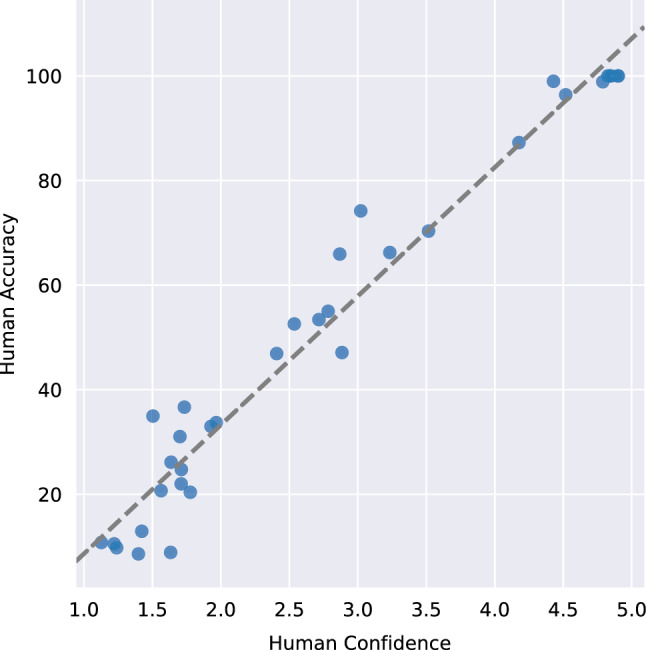
We found a linear correlation between human confidence scores and human accuracy. Humans are more confident of their performance on the easier transforms where they perform better. Networks show no such trend (Figure S2)

We analyzed the difference between the human and machine performance using multiple statistical tests. We tested both absolute performance on the same set of images and the observers’ confidence on these images. We used paired *t-*test statistic with 3 degrees of freedom (number of independent variables in our transform) to analyze the difference between networks and humans and found the difference between their performance to be significant (for numbers and transform specific tests, please see Table S4). We further used the *Pearson product-moment correlation* to see if the responses were correlated. We found the responses to be only weakly correlated in case of ResNet101, owing to its greater capacity and its performance to be marginally above chance in cases where the other two networks completely give up (for numbers and transform specific tests, please see Table S4). We also ran an *Ordinary Least Squares* (OLS) regression between human and network responses and found similar results (Tables S6, S7, S8, S9, S10).

To further examine our question about difference in strategy used by humans and machines for solving object recognition task, we statistically analyzed the confidence scores on same images classified by humans and networks. We found the *t-*test statistic to be consistent with our hypothesis about the two being different. (For numbers and transform specific tests, please see Table S5.) The correlation coefficient shows an overall negative correlation between the networks and humans (for numbers and transform specific tests, please see Table S5). While VOneResNet50 shows a nonnegative correlation, it is not statistically significant. VOneResNet50 also performed lowest overall. We saw a linear trend in relationship between confidence and accuracy for humans (Fig. 6). On tasks where humans performed with a higher accuracy, the confidence scores were high as well. We found the correlation coefficient for this trend to be over 98%, while the correlation between network confidences and their responses was well below 50%.

We plotted saliency maps from 0-shot experiments (please see §S3) because including them as part of the training process could introduce additional parameters which could potentially affect our analysis. We also calculated confidence score for networks as described in Gal and Ghahramani (2016) for a more equitable comparison to the human confidence that we collected in our psychophysics studies. Visualizing the weights of layers of these networks, however, does not show much and would not be a helpful comparison, given that our human experiments do not involve the use of eye-tracking devices or fMRI/EEG techniques. These are left for future studies.

**Fig. 7:**
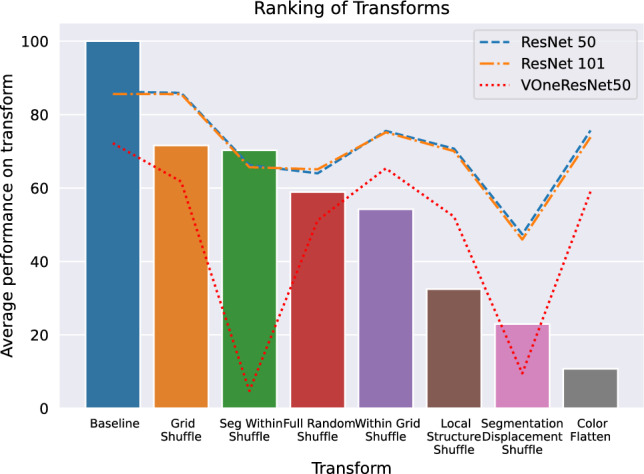
Average performance per transform for both humans and networks, sorted by human performance in descending order. Average performance for humans is shown as bars while that for networks is overlaid as lines. None of the networks agree with humans on a transform ranking. Individual rankings in Table S1

***Ranking of transforms*** We saw a linear correlation between human performance and human confidence scores (Fig. 6) and found that while they are related for humans, no such trend exists for machines. We next wanted to analyze human performance at a transform-level to compare the relationship across different transforms. While our transforms might look unrelated, they can be recreated by traversing a 3-dimensional space of independent variables, namely (1) block size, (2) shuffle probability and (3) moving the block to another location or not. We calculated a mean over human and network performance across individual transforms and ranked them in the order of decreasing human performance. Except baselines (no transforms), we did not find both humans and networks agreeing on assigning the same rank to any of the transforms. We found ResNet50 and ResNet101 to agree on most transforms, with the average performance also closely related. VOneResNet50 showed the most uneven trend compared to both humans and ResNets (Fig. 7), further illustrating its differences on a behavioral level. We present individual rankings per transform for both humans and machines (Table S1). Recall from Figs. 3 and 4 that variance between peak and average machine performance (not shown explicitly in the figure) as a function of block size is significantly high, while the variance between peak and average human performance (shown as horizontal gray line) is at the center of the range. This behavior further underscores differences between strategies used by humans and networks to solve our transforms. We present an analysis of parameter-level ranking for transforms in §S3.

## Discussion and Conclusion

Our work is inspired by the robustness of the human visual system in performing object recognition in the presence of extreme image distortions. We believe that humans use inductive biases and prior knowledge about the world to quickly switch between, or combine, bottom-up and top-down cues from the image features. Primate visual systems have feedback to better understand the visual scenes, while most object recognition networks rely on their feedforward behavior. Recent studies have highlighted the importance of recurrence to compete or exceed network performance on tasks that seem easy for humans (Linsley et al. 2021; Malik et al. 2021).

Unlike initial layers of CNNs that learn edges, contours, and textures, humans rely on an abstract concept of “object” representation and further add individual features linking it with an object’s category. The abstract concept of “an object” helps humans to learn about the characteristics of a given object and link it with the accompanying information about its environment. This happens at various levels of the human visual system (Carandini et al. 2005). During an object’s interaction with the environment, humans treat the object (independent of the class) as a whole individual entity, as opposed to parts of it interacting separately (Allison et al. 1994; Martin 2016; Keil and Müller 2010; Moon et al. 2021; Zmigrod and Hommel 2013; Levi et al. 1997). (The representation of the object being referred to here is atomic in nature. For entities with moving parts, individual parts can be treated as individual objects.) Most work in data transformation is on the augmentation side, wherein the added noise changes the pixel values. We wanted to keep the absolute pixel values intact in our transforms. Our transformations aim to test the understanding of “objectness” for the popular networks, swinging to the extreme ends of image manipulations.

Our results highlight that while CNNs learn representations in a feature specific manner, largely discounting the characteristic properties of the underlying object, humans try to learn the knowledge of features, building on top of objects (Perona and Malik 1990; Koenderink 1984, 2021). We find that networks are more affected by our segmentation transforms compared to our block transforms, further indicating their disconnect with human-like behavior. Networks have learned to solve tasks with noise as part of their training procedures to handle controlled adversarial attacks (Baradad Jurjo et al. 2021; Dapello et al. 2020), but struggle when the control is taken away. We filter humans and machines on the adversarial object recognition task, and are not creating systems that can break captchas (Noury and Rezaei 2020; Lin et al. 2018). We believe our work could be a step in that direction.

We show that machines perform better than humans on our “hard” transforms, while struggle to perform at par with humans on the “easy” transforms from a human perspective. We also show that this performance is highly correlated with confidence of selecting an object class for humans, while it is random at best for networks, highlighting the difference in strategies used by the two. Recent work on explaining V1 variance and building neural network blocks that simulate the neurophysiological data from visual cortex show promising results on controlled adversarial attacks, but still need more work to behaviorally perform like humans (Dapello et al. 2020, 2021). We show that the ranking at which these networks perform the task is very different from humans even at a coarse level. Recent work has applied random noise to pixels and intermediate layers to improve robustness to adversarial attacks (Liu et al. 2018). Including stochasticity to peripheral models has been proposed as a promising solution to learning more human-like representations (Dapello et al. 2021). We believe that robustness to such attacks should come from both input transformations and network architecture (Geirhos et al. 2018).

We demonstrate that human visual system employs more robust strategies in certain instances to solve the object recognition task, and highlight statistical differences in those strategies when compared to machines. Our novel transforms highlight a blind spot for the controlled adversarial training of networks. We hope these transforms can help with development/training of robust architectures simulating tolerance of primate visual system to deal with extreme changes in visual scenes often found in everyday settings.

## Limitation and future work

We used Imagenette [59], a subset of the larger Imagenet dataset (Deng et al. 2009) with 10 distinct and unrelated classes, due to compute limitations. We think using a larger subset could lead to more stable results, but will not affect the overall differences in patterns observed. We also asked for the confidence score from humans instead of calculating attention maps using fMRI/EEG techniques or using eye-tracking devices, due to limitations with participant recruiting and lack of an appropriate experimental infrastructure. We ran our human experiments in a standard way (Linsley et al. 2021; Frank et al. 2017) that should not affect the overall trends observed. We additionally filtered the participant responses with catch trials and median absolute deviation (Rousseeuw and Croux 1993). Not having attention data from humans limits our ability to correlate attention maps or feature weights from networks at a pixel-level.

### Supplementary Information

Below is the link to the electronic supplementary material.

Supplementary file 1 (pdf 3545 KB)

## Data Availability

Not available directly, but used for publication in aggregate form.

## Code Availability

No
