# Supplementary material for: Extreme image transformations affect humans and machines differently
Source: Biol Cybern. 2023 Jun 13;117(4-5):331–43. doi: 10.1007/s00422-023-00968-7 (PMC10600046; doi:10.1007/s00422-023-00968-7)
Supplement: Supplementary file 1 — (pdf 3545 KB) [file 422_2023_968_MOESM1_ESM.pdf]

Extreme Image Transformations Affect Humans and Machine Differently – Supplementary Information –

Appendix S1 Human Experiments Setup

We recruited 32 participants using Cloud Research's Connect platform for our psychophysics study, approved by Northeastern University's IRB (#22-10-09). The experiment was not time bound and could be completed at participants' own pace. The experiment was designed to take an average of 20-25 minutes. We compensated participants with a pro-rated minimum wage price of \$8 for their time. People with shorter and longer trial times were not compensated lesser or higher. We recorded the reaction time for all trials. After every trial, participants were redirected to a screen confirming their submission. They could continue by clicking the "Continue" button or pressing the spacebar. They were automatically redirected from the confirmation screen to the next screen in 2000ms. We also showed a "rest screen" after completion of every 10 trials, with a progress bar. The rest screen was shown only during main trials and not during practice trials. The time on rest screen was not recorded.

Experiment design

At the beginning of the experiment, the participants were shown an information screen guiding them about the significance of the experiment and what needs to be done. They could then click "Continue", which showed an instruction modal pop-up with instructions about what to do. They could view this instruction modal anytime during the experiment by clicking the button "Instructions" in the top right corner of their screens.

Participants were shown an image (baseline or transformed) along with ten object classes from the Imagenette dataset. They were asked to identify the object in the image and select the option closest to what they thought the object in the image was. They were also asked to rate their level of confidence on a scale of 1 through 5, ranging from least to most confident. They were given a feedback on their response in the form of correct or incorrect during practice trials but not during the main test trials. Each trial screen also had a short excerpt of instructions. Participants were shown a total of 11 practice trials and 102 test trials.

The class frequency of trial images was not explicitly balanced. In both practice and test trials, we did not focus on the object class as such, but rather on the transforms. We generated multiple images for every transform-hyperparameter pair across multiple classes and randomly selected 3 images for every transform-hyperparameter pair across all classes. After the selection, the same 102 images were shown to both networks and humans. The order of the display of images was randomly shuffled for both.

Software setup

The experiment used Python Flask for backend scripts and logic, and HTML, Bootstrap CSS framework and JavaScript for frontend. The form submission through keys and automatic redirections were done using jQuery on the user side. The server was run on HP Z200 workstation using 1 Intel(R) Xeon(R) CPU and 16 GB RAM.

Imagenette has 3-channel images of 320×320 pixel resolution. The images were sampled from test set at the original resolution for showing to human participants.

Filtering criteria

Data from cloud sourcing platforms can be noisy, with people identifying shortcuts to either quickly zip through the experiments or take very long on each trial. To eliminate such biases from the data, we excluded participants who either took a very long time, or failed on the baseline catch trials. To achieve our earlier filtering criteria, we removed participants for whom the response time did not exceed 2 median absolute deviations below the median, $median(X) - 2 * MAD(X)$, where MAD = median absolute deviation [83], a robust alternative to mean and standard deviation for identifying outliers.

Out of the 32 participants we recruited, 2 participants were filtered out by the above criteria. We removed all trials for the filtered out participants. Additionally, we recorded the accuracy of participants in practice trials and found all of them to perform with over 70% accuracy. We had also included catch trials in form of baseline and color flatten images, which are the easiest and hardest to classify respectively. None of the participants were found to be inattentive on the catch trials.

Appendix S2 Statistical Analysis of Human and Network Data

To test the significance of human responses compared to the data returned from the networks, we ran multiple statistical tests, trying to understand if the performance was significant, and if the strategies used by the networks were similar in any way. We used t-test statistic, correlation coefficient and Ordinary Least Squares regression (OLS) to fit human and network data. We used `statsmodels` [84] library in python for our analysis. For t-test, we considered 3 degrees of freedom based on the number of independent variables that could be altered to get our transforms – block size, probability of shuffle, and moving the block to another position or not.

Appendix S3 Ranking of Transforms

Our transforms are based on three independent variables – i) Block size (or number of segments in case of segmentation shuffles), ii) Probability of individual pixel shuffle and iii) Moving the block/region to another location or

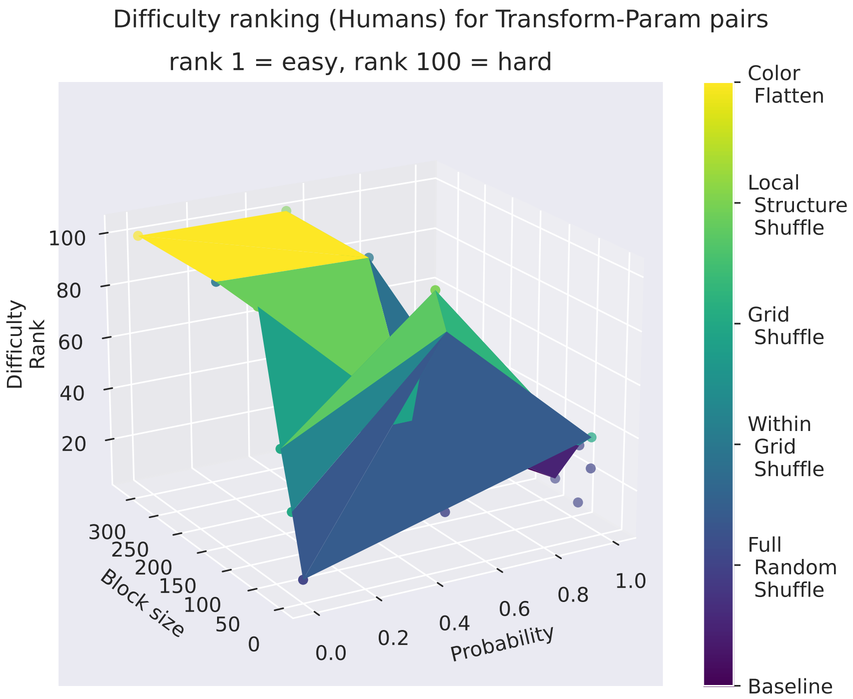

Fig. S1 Ranking of Transforms: Difficulty rank as a function of block size and probability for human observers. Higher rank is harder for humans. The surface looks like a bird flapping wings, indicating lower difficulty for a few cases, but shows an overall increase in difficulty with a change in independent variables. See text for details

not. Traversing this 3-dimensional space leads to a wide variety of variations in the visual perception of objects for humans and machines. To link it all together, we ranked the transforms by sorting collective human performances on the transformation-parameter pairs. We calculate an overall ranking of all the transforms in Table S2 and plot it based on their probability and block sizes (10% accuracy is treated as chance). Difficulty rank is calculated by $100 - accuracy$ across all images in the respective transformation-parameter pair (higher rank is harder). For a discussion about coarse transform-level ranking, please see §6.

The influence of independent variables on the performance of humans is shown by the surface in Fig S1. The shape of surface looks like that of a bird flapping its wings. We found the performance to be inversely related to the increase in probability of shuffle, and directly related to an increase in block size, in line with what has been observed in the field [32, 85–89] We observed an opposite trend in case of networks. The conditions where humans performed well, were hard for networks and vice-versa (Table S5). Human responses are treated as independent variables.

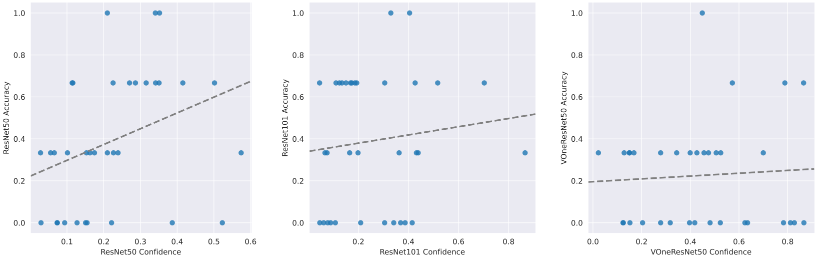

Fig. S2 Correlation between network confidence and accuracy. (L-R) ResNet50, ResNet101, VOneResNet50. We see that the trend is not linear (as in case of humans, Fig 6).

While we observe a global upward trend in difficulty of transforms, we also observe certain outliers where complexity decreases for certain transforms. This is specially true in cases like Within Grid Shuffle (probability = 0.5), Local Structure Shuffle (probability = 0.5 and block size = 160) [shown in turquoise and green], where the larger block sizes aid in feature identification.

Correlation in network responses

Similar to the correlation analysis between human accuracy and human confidence (Figure 6), we tried linear curve fitting for network accuracy v/s network confidence. While humans show a clearly linear trend, networks do not show any such correlations (Figure S2).

Insights into how networks classify images

To further substantiate our claim that networks cannot generalize to novel objects without the need for finetuning — while humans can recognize and generalize in the wild — we performed 0-shot experiments with ResNet50, ResNet101 and VOneResNet50. We started with Imagenet [33] weights for all 3 networks and evaluated them on the Hymenoptera dataset [90, 91] containing bees and ants. We found that none of the 3 networks were able to classify the bees and ants correctly on baseline and transforms alike. We instead found that networks worked well with the low-level feature of color contrasts and showed high activity where the objects stood out from the background, as shown by their respective saliency maps (Figures S3, S4). In transforms where the contrasts between foreground and background are not strong, or where the pixels are more randomly shuffled, the activity in these networks is more evenly distributed. In case of segmentation shuffle transforms, the networks do not seem to trace the contours of segmentation.

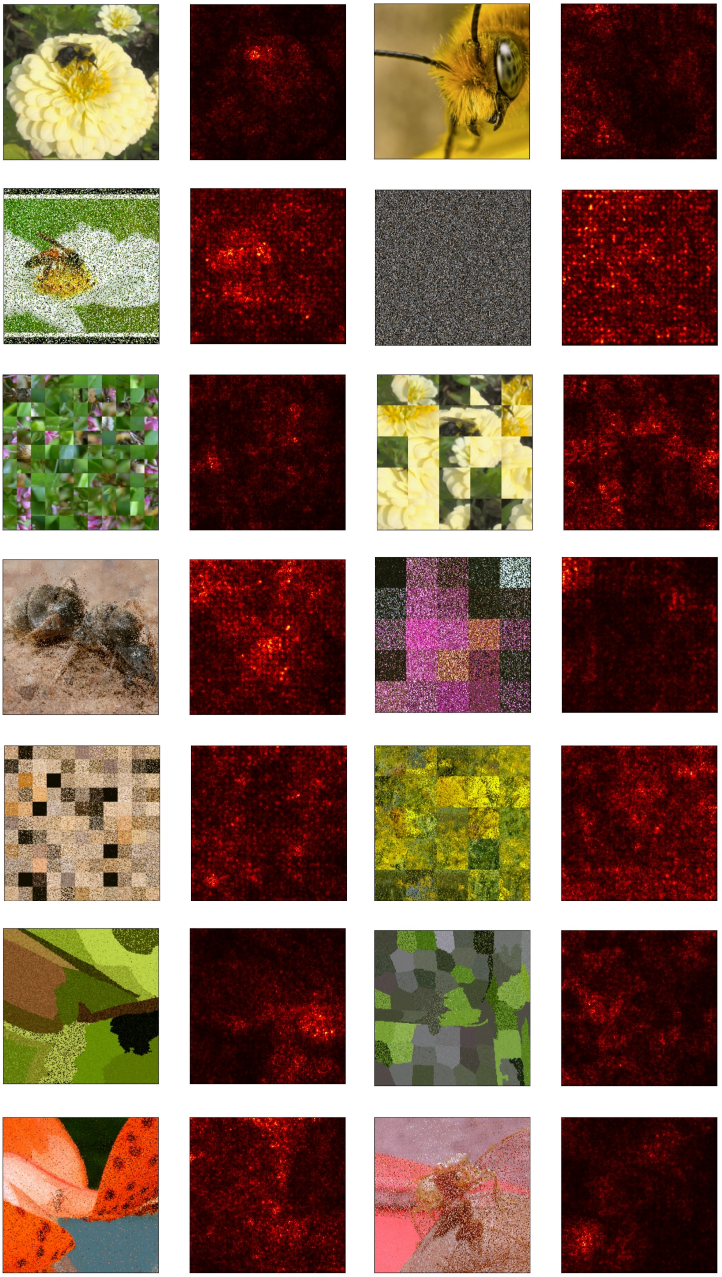

Fig. S3 Extreme Image Transformed images and saliency maps from Imagenet pretrained ResNet50 evaluated on Hymenoptera dataset. Images **L-R and T-B** are baseline, baseline, Full Random Shuffle($p = 0.5$ and $p = 1.0$), Grid Shuffle($b = 20$ and $b = 40$), Within Grid Shuffle($b = 20, p = 0.5$ and $b = 40, p = 1.0$), Local Structure Shuffle($b = 20, p = 1.0$ and $b = 40, p = 0.5$), Segmentation Displacement Shuffle($b = 16$ and $b = 64$), and Segment Within Shuffle($b = 8, p = 0.5$ and $b = 16, p = 0.5$). The saliency maps clearly show that ResNet50 focuses mostly on the contrast of patches and does not explicitly attend to the object in focus.

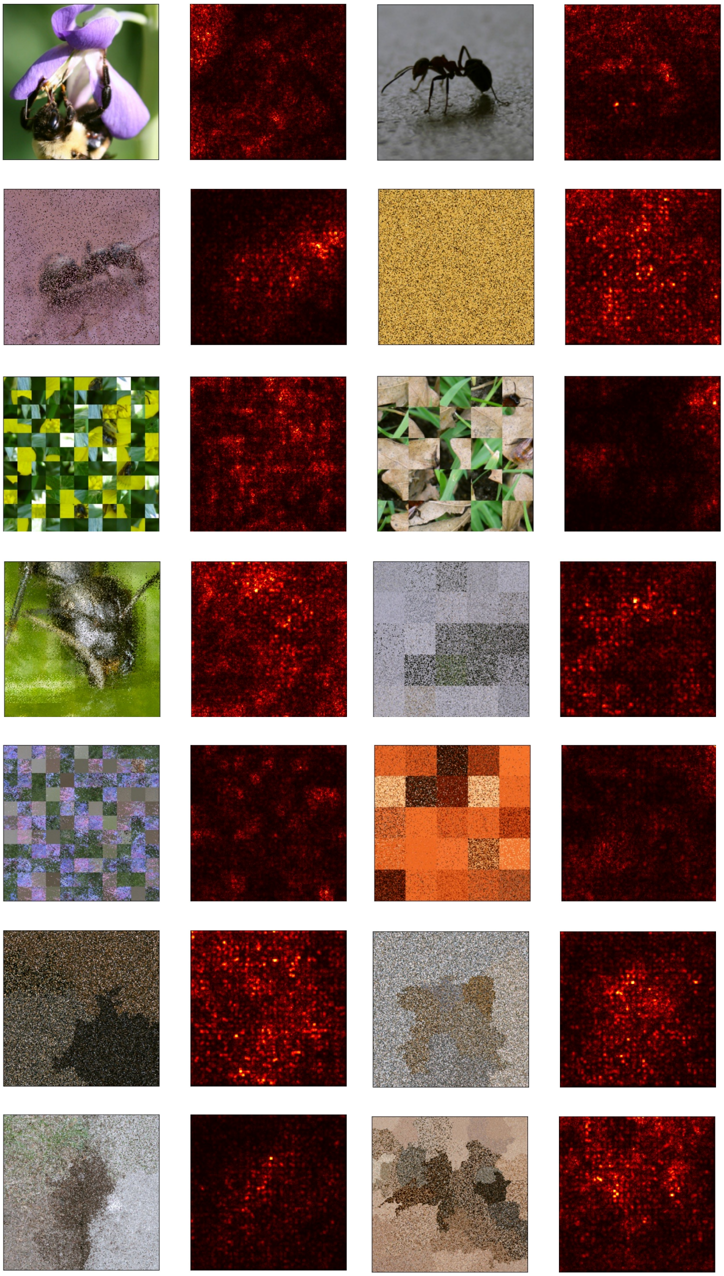

Fig. S4 Extreme Image Transformed images and saliency maps from Imagenet pretrained ResNet101 evaluated on Hymenoptera dataset. Images **L-R and T-B** are baseline, baseline, Full Random Shuffle($p = 0.5$ and $p = 1.0$), Grid Shuffle($b = 20$ and $b = 40$), Within Grid Shuffle($b = 20, p = 0.5$ and $b = 40, p = 1.0$), Local Structure Shuffle($b = 20, p = 0.5$ and $b = 40, p = 1.0$), Segmentation Displacement Shuffle($b = 8$ and $b = 64$), and Segment Within Shuffle($b = 8, p = 0.5$ and $b = 64, p = 1.0$). The saliency maps clearly show that ResNet101 focuses mostly on the contrast of patches and does not explicitly attend to the object in focus. The area covered in saliency maps is higher than that compared to ResNet50, mostly due to the higher number of parameters in the network.

Table S1 Transform-level Ranking for Humans and Networks, sorted by Human performance. Higher rank is harder.

Transform	Human	ResNet50	ResNet101	VOne
Baseline	1	1	1	1
Full Random Shuffle	2	2	2	3
Grid Shuffle	3	6	6	8
Within Grid Shuffle	4	7	7	6
Local Structure Shuffle	5	4	3	2
Segmentation Displacement Shuffle	6	5	5	5
Segmentation Within Shuffle	7	8	7	7
Color flatten	8	3	4	4

Table S2 Test accuracy for humans and networks trained on Imagenette dataset with Block transforms

Transform	P	Grid Size	Accuracy (in %)			
			ResNet50	ResNet101	VOne	Human
Baseline			66.67	33.33	0	100
Full Random Shuffle	0.5		66.67	66.67	0	100
	0.8		0	0	66.67	64.06
	1.0		33.33	0	33.3	12.82
Grid Shuffle		20x20	0	0	0	32.99
		40x40	33.33	33.33	33.33	53.42
		80x80	66.67	66.67	33.33	100
		160x160	66.67	66.67	66.67	100
Within Grid Shuffle	0.5	20x20	33.33	33.33	0	87.25
		40x40	66.67	66.67	0	100
		80x80	0	0	0	47.11
		160x160	66.67	66.67	66.67	98.89
	1.0	20x20	33.33	33.33	33.33	24.74
		40x40	66.67	66.67	66.67	31.03
		80x80	33.33	66.67	33.33	34.96
		160x160	100	66.67	0	9.78
Local Structure Shuffle	0.5	20x20	1	1	33.33	20.39
		40x40	66.67	66.67	1	55
		80x80	0	33.33	0	46.91
		160x160	66.67	66.67	33.33	70.33
	1.0	20x20	1	1	0	36.67
		40x40	0	0	0	8.89
		80x80	33.33	33.33	0	12.93
		160x160	33.33	0	33.33	8.6
Color Flatten			66.67	66.67	33.33	10.78

Table S3 Test accuracy for humans and networks trained on Imagenette dataset with Segmentation transforms

Transform	P	Segments	Accuracy (in %)			
			**ResNet50**	**ResNet101**	**VOne**	**Human**
Segmentation Displacement Shuffle		8	33.33	33.33	33.33	22
		16	0	0	0	20.69
		64	0	0	0	26.14
Segmentation Within Shuffle	0.5	8	33.33	0	33.33	65.93
		16	33.33	66.67	33.33	96.39
		64	33.33	66.67	33.33	98.98
	1.0	8	0	33.33	0	52.58
		16	0	0	0	74.19
		64	0	0	0	33.71

Table S4 *t*-test statistic and Correlation Coefficient between human and network responses. Networks are ResNet**50**, ResNet**101**, and **VOne**ResNet50 in that order. Human responses are averaged across all participants for the mentioned transforms. p-values are indicated in the parentheses below the statistic.

Transform	*t*-test Statistic			Correlation Coefficient		
	**50**	**101**	**VOne**	**50**	**101**	**VOne**
All transforms	-1.57 (0.1217)	-1.46 (0.1217)	-4.55 (0.0002)	0.17	0.32	0.16
Full Random Shuffle	-0.79 (0.4777)	-1.07 (0.3459)	-1.29 (0.2951)	0.37	0.79	-0.79
Grid Shuffle	-1.29 (0.2456)	-1.29 (0.2456)	-1.76 (0.1307)	0.98	0.98	0.81
Within Grid Shuffle	-0.25 (0.8044)	-0.27 (0.7896)	-1.78 (0.0976)	-0.06	0.06	-0.03
Local Structure Shuffle	1.07 (0.3053)	1.07 (0.3053)	-1.52 (0.6213)	0.28	0.47	0.28
Segmentation Displacement Shuffle	-1.05 (0.3987)	-1.05 (0.3987)	-1.05 (0.3987)	-0.29	-0.29	-0.29
Segmentation Within Shuffle	-4.22 (0.0022)	-2.32 (0.0319)	-4.22 (0.0022)	0.73	0.77	0.73

Table S5 *t*-test statistic and Correlation Coefficient between human and network confidence. Networks are ResNet**50**, ResNet**101**, and **VOne**ResNet50 in that order. Human confidence is averaged across all participants for the mentioned transforms. p-values are indicated in the parentheses below the statistic.

**Transform**	***t*-test Statistic**			**Correlation Coefficient**		
	**50**	**101**	**VOne**	**50**	**101**	**VOne**
All transforms	-11.00 (1.0492E-12)	-10.78 (1.4316E-12)	-9.95 (8.8715E-12)	-0.06	-0.17	0.22
Full Random Shuffle	-2.77 (0.1084)	-2.68 (0.1152)	-2.67 (0.1166)	0.33	-0.74	0.65
Grid Shuffle	-4.48 (0.0202)	-4.57 (0.0196)	-4.14 (0.0253)	0.95	0.17	0.25
Within Grid Shuffle	-4.77 (0.0019)	-4.58 (0.0023)	-3.90 (0.0057)	-0.11	-0.02	0.43
Local Structure Shuffle	-6.53 (0.0002)	-6.10 (0.0003)	-5.90 (0.0005)	-0.21	-0.40	0.24
Segmentation Displacement Shuffle	-25.56 (0.0000)	-12.14 (0.0024)	-14.23 (0.0010)	0.68	-0.04	0.01
Segmentation Within Shuffle	-7.11 (0.0007)	-7.44 (0.0007)	-7.26 (0.0000)	-0.69	0.54	-0.02

Table S6 OLS for all data (baseline and transforms)

**Dep. Variable:**	y	**R-squared (uncentered):**	0.903
**Model:**	OLS	**Adj. R-squared (uncentered):**	0.890
**Method:**	Least Squares	**F-statistic:**	69.87
**Date:**	Sat, 26 Nov 2022	**Prob (F-statistic):**	9.12e-15
**Time:**	13:13:29	**Log-Likelihood:**	-8.5703
**No. Observations:**	34	**AIC:**	25.14
**Df Residuals:**	30	**BIC:**	31.25
**Df Model:**	4		

	**coef**	**std err**	**t**	**P>** t	**[0.025**	**0.975]**
**Humans**	0.1658	0.035	4.713	0.000	0.094	0.238
**ResNet50**	0.8358	0.396	2.111	0.043	0.027	1.644
**ResNet101**	0.5925	0.334	1.775	0.086	-0.089	1.274
**VOneResNet50**	0.2324	0.268	0.867	0.393	-0.315	0.780

**Omnibus:**	2.006	**Durbin-Watson:**	0.975
**Prob(Omnibus):**	0.367	**Jarque-Bera (JB):**	1.347
**Skew:**	-0.229	**Prob(JB):**	0.510
**Kurtosis:**	2.139	**Cond. No.**	23.6

Table S7 OLS for Within Grid Shuffle

**Dep. Variable:**	y	**R-squared (uncentered):**	0.975
**Model:**	OLS	**Adj. R-squared (uncentered):**	0.950
**Method:**	Least Squares	**F-statistic:**	39.38
**Date:**	Sat, 26 Nov 2022	**Prob (F-statistic):**	0.00181
**Time:**	13:13:29	**Log-Likelihood:**	3.4419
**No. Observations:**	8	**AIC:**	1.116
**Df Residuals:**	4	**BIC:**	1.434
**Df Model:**	4		

	**coef**	**std err**	**t**	**P>** t	**[0.025**	**0.975]**
**Humans**	-0.0244	0.067	-0.366	0.733	-0.209	0.160
**ResNet50**	-0.2386	0.693	-0.344	0.748	-2.164	1.686
**ResNet101**	-0.1106	0.473	-0.233	0.827	-1.425	1.204
**VOneResNet50**	1.5680	0.568	2.760	0.051	-0.009	3.145

**Omnibus:**	1.314	**Durbin-Watson:**	1.668
**Prob(Omnibus):**	0.518	**Jarque-Bera (JB):**	0.718
**Skew:**	0.310	**Prob(JB):**	0.698
**Kurtosis:**	1.669	**Cond. No.**	37.7

Table S8 OLS for Local Structure Shuffle

**Dep. Variable:**	y	**R-squared (uncentered):**	0.992
**Model:**	OLS	**Adj. R-squared (uncentered):**	0.984
**Method:**	Least Squares	**F-statistic:**	125.4
**Date:**	Sat, 26 Nov 2022	**Prob (F-statistic):**	0.000187
**Time:**	13:13:29	**Log-Likelihood:**	8.0076
**No. Observations:**	8	**AIC:**	-8.015
**Df Residuals:**	4	**BIC:**	-7.698
**Df Model:**	4		

	**coef**	**std err**	**t**	**P>** t	**[0.025**	**0.975]**
**Humans**	0.0617	0.064	0.958	0.392	-0.117	0.240
**ResNet50**	0.3270	0.695	0.471	0.662	-1.602	2.256
**ResNet101**	0.5928	0.460	1.288	0.267	-0.685	1.870
**VOneResNet50**	1.0817	0.251	4.313	0.013	0.385	1.778

**Omnibus:**	0.077	**Durbin-Watson:**	1.757
**Prob(Omnibus):**	0.962	**Jarque-Bera (JB):**	0.153
**Skew:**	0.109	**Prob(JB):**	0.926
**Kurtosis:**	2.358	**Cond. No.**	42.4

Table S9 OLS for Segmentation Within Shuffle

**Dep. Variable:**	y	**R-squared (uncentered):**	0.987
**Model:**	OLS	**Adj. R-squared (uncentered):**	0.961
**Method:**	Least Squares	**F-statistic:**	38.11
**Date:**	Sat, 26 Nov 2022	**Prob (F-statistic):**	0.0257
**Time:**	13:13:29	**Log-Likelihood:**	4.5261
**No. Observations:**	6	**AIC:**	-1.052
**Df Residuals:**	2	**BIC:**	-1.885
**Df Model:**	4		

	**coef**	**std err**	**t**	**P>** t	**[0.025**	**0.975]**
**Humans**	0.0982	0.107	0.914	0.457	-0.364	0.560
**ResNet50**	0.4798	0.570	0.841	0.489	-1.974	2.933
**ResNet101**	0.3946	2.177	0.181	0.873	-8.973	9.762
**VOneResNet50**	3.5181	2.264	1.554	0.260	-6.225	13.261

**Omnibus:**	nan	**Durbin-Watson:**	2.906
**Prob(Omnibus):**	nan	**Jarque-Bera (JB):**	0.324
**Skew:**	0.467	**Prob(JB):**	0.850
**Kurtosis:**	2.348	**Cond. No.**	96.8

Table S10 OLS for all segmentation shuffles

**Dep. Variable:**	y	**R-squared (uncentered):**	0.959
**Model:**	OLS	**Adj. R-squared (uncentered):**	0.925
**Method:**	Least Squares	**F-statistic:**	28.91
**Date:**	Sat, 26 Nov 2022	**Prob (F-statistic):**	0.00119
**Time:**	13:28:32	**Log-Likelihood:**	1.5554
**No. Observations:**	9	**AIC:**	4.889
**Df Residuals:**	5	**BIC:**	5.678
**Df Model:**	4		

	**coef**	**std err**	**t**	**P>** t	**[0.025**	**0.975]**
**Humans**	0.1604	0.062	2.575	0.050	0.000	0.320
**ResNet50**	0.7505	0.695	1.080	0.330	-1.036	2.537
**ResNet101**	-0.7065	2.035	-0.347	0.743	-5.938	4.525
**VOneResNet50**	2.8102	2.297	1.224	0.276	-3.093	8.714

**Omnibus:**	1.418	**Durbin-Watson:**	1.607
**Prob(Omnibus):**	0.492	**Jarque-Bera (JB):**	0.970
**Skew:**	0.597	**Prob(JB):**	0.616
**Kurtosis:**	1.922	**Cond. No.**	97.5
